# Impact of non-surgical OR time on efficiency and costs with Hugo™ RAS

**DOI:** 10.1007/s11701-025-02731-5

**Published:** 2025-09-05

**Authors:** Christopher Hirtsiefer, Roman Herout, Sherif Mehralivand, Susanne Oelkers, Claudia Franz, Christian Thomas, Martin Baunacke

**Affiliations:** 1 Department of Urology, Medical Faculty Carl Gustav Carus, TU Dresden, Fetscherstr. 74, 01307 Dresden, Germany; 2 Vancouver Prostate Centre, Department of Urologic Sciences, Medical Faculty, University of British Columbia, Vancouver, Canada

**Keywords:** Robotic-assisted surgery, Radical prostatectomy, daVinci, HugoRAS, Surgical efficiency, Operative time

## Abstract

Robot-assisted surgery is increasingly preferred. New systems such as the Hugo™RAS enter the market, offering different pricing and modular architecture. While daVinci systems dominate U.S. practice, the HugoRAS system is undergoing early implementation. Understanding non-surgical, patient-independent time (NonSPIT)—a major driver of cost and efficiency—is crucial for institutions considering adoption. We conducted a prospective, single-center study in Germany, comparing perioperative times of robotic pelvic procedures (RPP) using newly introduced HugoRAS and preestablished daVinci Xi, and retrospectively of open pelvic procedures (OPP). NonSPIT, defined as setup, docking, undocking, and system cleanup, was recorded and analyzed with respect to personnel experience. The resulting costs were estimated. 167 RPP using HugoRAS (*n* = 144) and Xi (*n* = 23) were included and supplemented with 20 historical OPP. NonSPIT accounted for 40.0% (HugoRAS) and 36.7% (Xi) of total procedure time, compared to 31% in OPP. HugoRAS required 13 more minutes of NonSPIT than Xi (94.3 vs. 81.6 min; *p* < 0.05–OPP: 68.4 min). This overall disadvantage was nullified after 10–15 cases per scrub nurse. 77.1% of NonSPIT difference was caused by setup, where RPP showed longer preparation times than OPP (45.1 and 33.5 min vs. 27.4 min). HugoRAS setup was slower than Xi for unexperienced (48.0 vs. 33.5 min; *p* < 0.01) and experienced teams (44.5 vs. 33.5 min; *p* < 0.01). The projected additional time costs of NonSPIT in HugoRAS RPP amounts to $865.55 and $1325.95 per procedure compared to Xi and OPP, respectively. HugoRAS’ cost of learning is estimated at $7017.87 per nurse until efficiency of an established Xi system is achieved. In the U.S. context, HugoRAS may initially be less time-efficient than daVinci Xi. However, this gap closes after approximately 15 procedures, leaving behind only a small disadvantage in system preparation and making HugoRAS an economical alternative—particularly given its lower per-case instrumentation costs.

**Supplementary Information:**

The online version contains supplementary material available at 10.1007/s11701-025-02731-5.

## Introduction

Robot-assisted surgery (RAS) has become a hallmark of modern urologic care, particularly in the context of robotic radical prostatectomy (RARP) [1]. Patients perceive robotic techniques as preferably [2] even in the absence of clear evidence of superior oncological or functional outcomes [3]. Recent multicentric data show that patients are willing to travel further to undergo robotic rather than open surgery [4]. This patient preference for RARP over open prostatectomy (ORP) is backed by lower cumulative healthcare cost in the first postoperative year, but not necessarily by lower cost of index hospitalization, which are driven by cost for robotic systems [5].

With the expiration of core patents held by daVinci’s manufacturer Intuitive (Intuitive Surgical, Sunnyvale, CA, USA) in 2019 [6], new robotic platforms have entered the market, offering alternative technical features and pricing models [7]. Among these, the Hugo™ Robotic Assisted Surgery (RAS) System by Medtronic (Medtronic, Minneapolis, MN, USA) (HugoRAS) has emerged as a modular, multi-cart system, in contrast to the single-cart architecture of the da Vinci^®^ system [8]. While HugoRAS has gained regulatory approval and clinical adoption in Europe since 2022 and in Germany since 2023, its use in the United States remains in early phases with FDA approval for urologic indications still pending in April 2025 [9].

Despite similar console ergonomics, HugoRAS is assumed to require additional time and coordination for setup due to its decentralized cart architecture [8]. Previous work suggested longer docking times than in daVinci Xi (Xi) during the initial phase of system implementation. This effect was shown to be rooted in the performance of the unsterile scrub nurse moving the patient carts [10]. Although laser-assisted docking can mitigate early inefficiencies in positioning [10], docking represents only a small portion of the economically relevant OR time spent per procedure. Another study of 30 cases has also shown system preparation to be more time-consuming in a newly acquired HugoRAS compared to daVinci and failed to observe a significant improvement over time [11].

In a time of increasing economic pressure and staff shortages caused by a shrinking number of trainees [12], surgical efficiency has gained renewed importance. The cost of acquisition, instruments and maintenance per procedure have recently been reported to differ approximately 250 € (USD equivalent when published 2024: $270) between the two systems (€2246 for DaVinci, €1995 for HugoRAS; equivalent to $2426 and $2155) [13]. With a minute of OR time ranging from $25 to $133/min [14–16], recently summarized to an average of $ 46.04/min [17], even small improvements of process durations are highly relevant.

This study aims to assess and compare time resources required for robotic-assisted radical prostatectomy using a locally established Xi and a newly introduced HugoRAS. By analyzing perioperative time metrics across different stages of implementation and operator experience, we aim to provide insights on patient-independent time expenditures relevant to institutions transitioning from da Vinci to Hugo or enlarging their robotic portfolio.

## Methods

### Study setting

This single center, prospective observational study recorded time measurements of robotic assisted pelvic procedures (RPP) using the Xi and the HugoRAS systems. For comparison, historic time data of open pelvic procedures (OPP) was gathered retrospectively.

Surgeries took place from October 2023 to January 2025 (HugoRAS), October 2023 to April 2025 (Xi) and January to March 2018 (OPP) at the Department of Urology of the University Hospital of Dresden, Germany. For HugoRAS, recorded procedures represent the first consecutive RPP performed after introduction of the system at this center. Dresden was the first Urologic Center to introduce this system in Germany. For Xi, reference times were measured infrequently, where organizationally feasible, and equipment not used in Hugo procedures. No deliberate selection per case, patient, staff or daytime was implemented. OPP data collection was retrieved from a period of high OPP caseload in the center.

**Fig. 1:**
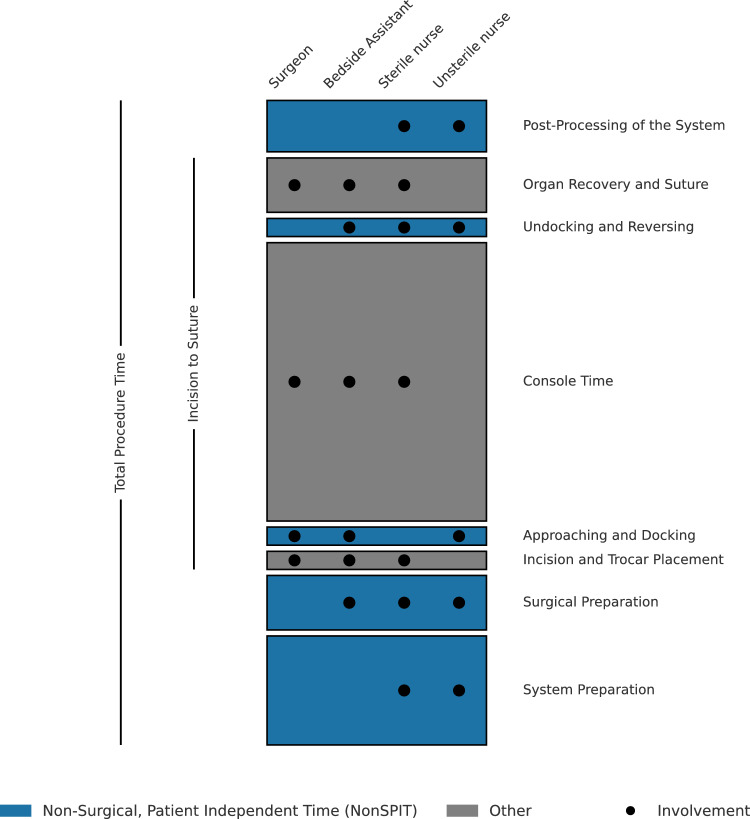
Showing relevant processes during robotic pelvic procedures and staff involvement

All personnel operating the systems was experienced in RPP, with the Xi deployed in the department in 2022 and predecessors used on site since 2006. Personnel operating the systems consists of a urologic surgeon, a bedside assistant (urologic resident) and two scrub nurses. To assess personal effects of learning with the newly implemented HugoRAS system, staff attendance was recorded. The unsterile scrub nurse was responsible for positioning the patient cart(s) and connecting wiring, while surgeon and bedside assistant performed docking of the systems (Fig. 1).

### Measurements

Time measurement of predefined periods (Fig. 1) was implemented with “Now Then Time Tracking Pro” Version 2.3.1(Angry Aztec Ltd, Perth, Scotland) for iPad OS. For comparative OPP data, time periods were taken from the center’s digital records.

The following time periods were recorded:

- System Preparation including powering up and connecting the robotic system, setting up sterile tables, gathering, unwrapping and preparing sterile equipment for use, covering robotic arms in sterile foil and prepositioning them. Timing begins with activating the robotic system and ends with anesthesiologic clearance to position the patient or beginning of surgical standby for the latter.
- For OPP, System Preparation is retrieved from the center’s digital records, where preparation starts with setting up sterile tables and ends with the patient entering the OR.
- Surgical Preparation, including positioning of the patient, surgical site preparation, preparation of suction and electrical and optical wiring. Timing begins with the start of positioning, includes skin antisepsis and ends with the beginning of the team-time out.
- Incision and Trocar Placement, including marking incision sites (if applicable), primary incision or puncturing, CO2 insufflation, insertion of first trocar, explorative laparoscopy, optically controlled skin incision for and placement of four additional trocars, removal of adhesions where needed for trocar placement and change of insufflation to assistant trocar. Timing begins with the first mark or the first incision and ends with surgeon’s clearance to approach robotic system.
- Approaching and docking, additionally including the adjustment of arm angles, connecting and inserting instruments. Timing begins with the release of the (first) patient cart’s break and ends with the surgeon’s approval of successful docking.
- Undocking and Reversing, including removing instruments, undocking arms, and reversing the patient cart(s). Timing starts with the surgeon’s clearance to undock and ends with stop of patient cart(s) in distant position.
- Organ Recovery and Suture, including laparoscopically retrieving the recovery bag’s thread to the outside, removing all trocars, widening the central trocar wound, recovering the prostate, hemostasis if applicable, multilayer suture of the central trocar wound, single- or double-layer sutures of other trocar wounds. Timing starts with inserting the laparoscopic forceps and ends with finalization of the last suture.
- Post-Processing of the System, including wound dressing, uncovering the patient, uncovering and sanitizing the robotic system, flushing or discarding robotic instruments, placing all reusable instruments back for autoclaving and discarding of single use items. Timing starts subsequently after finishing the last suture and ends with scrub nurses finishing material handling for this procedure, independent of length of possibly prolonged anesthesia. As HugoRAS system required a full system shutdown in between procedures, the moment of shutdown was used as endpoint if superseding material handling.

For OPP, this time was retrieved from center’s digital records, starts at the same time and ends with scrub nurses leaving the OR.

Additionally, Incision-to-Suture and Total Procedure Times were separately retrieved from digital records for all procedures. Total Procedure Time starts with system and room preparation and ends with the beginning of room cleaning. Inaccuracies caused by undefined waiting time were labeled as Unaccounted Time in Graphics. By design, no OPP data is available for time periods c, d, e, and f.

### Data processing

Non-surgical, patient independent time in total (*NonSPIT*) was calculated as the sum of System Preparation, Surgical Preparation, Approaching and Docking, Undocking and Reversing, as well as Post-Processing of the System. Deviating only in method of calculation, but not in result, NonSPIT for OPP is the subtraction of Incision-to-Suture Time from Total Procedure Time.

For analysis of learning effects, procedures performed by each unsterile scrub nurse were numbered individually to represent each nurse’s experience at any time. Unsterile scrub nurses were selected for deeper analysis in line with previous Propensity Score findings of them being the pacesetters of docking [10]. Individual HugoRAS procedure count was divided in two groups (unexperienced: individual procedure count 1–5, experienced: count 10–15) to compare newly adapting and experienced personnel. Averages of individual procedure count were weighed according to data availability, as scrub nurses participated in different total numbers of procedures. With the Xi system having been in place for years ahead, procedures were not counted for Xi.

To enhance reliability of data and reduce impact of extreme values in individual cases, measurements exceeding two standard deviations (SD) were excluded. Obvious software or user errors in timekeeping were excluded by introducing minimal cut-off values for Incision and Trocar Placement (< 3 min), Approaching and Docking (< 1 min), Undocking and Reversing (< 1 min) and Post-Processing of the System (< 4 min).

To estimate overall differences in operating costs stemming from time differences, simulations of time loss and financial (dis-)advantages over time will be set up. Calculations will be based on the previously reported cost of $ 46.04 per minute of OR time.

Unconnected t-tests were used for statistical analysis. *p* < 0.05 was considered to indicate significance. Due to the differences in data acquisition between OPP and RPP, statistical tests were only conducted within the robotic cohort. Statistical analysis was performed using R Studio (Version 2024.09.0 + 375) (Posit Software, Boston, MA, USA) [18]. Key analyses are repeated without exclusion of outliers to control for the impact of extreme values on statistical findings. Additional delta-analyses without statistical testing may be included to investigate influence on NonSPIT. This is an observational study. Ethical approval was obtained (17.08.2023/No. BO-EK-273062023).

## Results

A total of *N* = 167 pelvic robotic assisted procedures (HugoRAS, *N* = 144; Xi, *N* = 23) were timed and time data of 20 OPP extracted from the centre’s digital records. Of the procedures performed with HugoRAS, 136 (94.4%) were RARPs, seven (4.9%) were simple prostatectomies and one (0.7%) was a vesicu-vaginal fistula repair. All OPP and all Xi procedures were ORP/RARP.

From a total of 1249 single time measurements, 70 were excluded from analysis due to missing input. Further data processing led to the exclusion of 56 measurements exceeding 2 SD cutoffs and another 43 single measurements undercutting minimum periods (Supplementary Table 1).

### System comparison

The average total procedure time of robotic procedures was 233.6 min (149.9 min–321.3 min) with OPP referencing averaging to 220.8 min (170.8 min–280.0 min).

Within the robotic cohort, Total Procedure Time accounted for 235.2 min (149.9 min–321.3 min) in HugoRAS and 222.6 min (157.0 min–306.0 min) in Xi, with no detectable difference (*p* = 0.27).

NonSPIT represented 40.0% of Total Procedure Time in HugoRAS (94.3 min, Total: 235.2 min) and 36.7% in Xi (81.6 min, Total: 222.6 min). Taking 13 min more, overall NonSPIT was significantly longer for HugoRAS compared to Xi (94.3 vs. 81.6 min; *p* < 0.05).

**Fig. 2:**
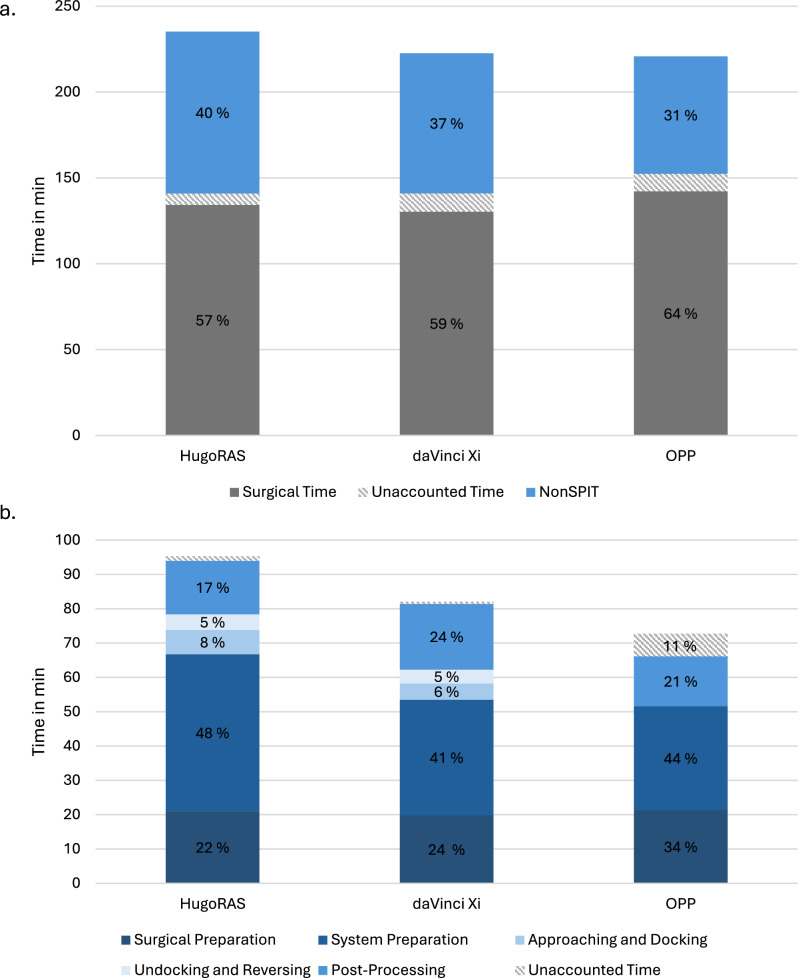
Showing the partitioning of **a** total procedure time and **b** NonSPIT. Surgical is calculated as Incision-to-Suture time subtracted by Approaching, Docking, Undocking and Reversing as accounted for in NonSPIT. *NonSPIT* non-surgical, patient independent time in total; *Hugo RAS* Hugo™ Robotic Assisted Surgery (RAS) System by Medtronic (Medtronic, Minneapolis, MN, USA); *daVinci Xi* Da Vinci Xi (Intuitive Surgical, Sunnyvale, CA, USA; *OPP* open pelvic procedures

NonSPIT in retrospective OPP reference data represented 31.0% of its Total Procedure Time (68.4 min; 45.0 min–179.0 min) (Fig. 2a).

Investigating components of NonSPIT, System Preparation represented the largest portion of NonSPIT for all setups (Xi: 49%, HugoRAS 63%, OPP 67%) (Fig. 2b). Significantly slower performance in HugoRAS were captured for System Preparation (45.1 vs. 33.5 min; *p* < 0.01) as well as Approaching and Docking (7.0 vs. 4.7 min; *p* = 0.02) (Table 1). An additional multiple linear regression showed no influence on NonSPIT by nerve sparing (onesided: *p* = 0.66, twosided: *p* = 0.43) or lymphadenectomy (performed: *p* = 0.86).

**Table 1:** Time measurements per group

Time variable	HugoRAS^a^ (*N* = 144)		daVinci Xi^b^ (*N* = 23)	OPP^c^ (*N* = 20)	p*
	All (*N* = 144)	Experienced scrub nurses (*N* = 28/144)			
System preparation (in min) *N* = 177	45.1 (16.5–73.2)	44.5 (16.5–65.0)	33.5 (14–54)	27.4 (15–65)	**< .01**
Surgical preparation (in min) *N* = 181	21.0 (9–38)	22.0 (12.5–37.2)	19.8 (11–37)	21.3 (10–38)	.40
Incision and trocar placement (in min) *N* = 149	8.0 (3.1–19.2)	7.8 (3.1–13.7)	7.0 (4–19)	n.a	.28
Approaching and docking (in min) *N* = 156	7.0 (1.8–25.3)	6.9 (1.9–17.0)	4.7 (1.1–14)	n.a	**.02**
Undocking and reversing (in min) *N* = 143	4.5 (1.1–14.9)	5.6 (1.4–14.3)	4.0 (1.0–11.0)	n.a	.35
Incision to suture (in min) *N* = 168	153.9 (89.6–231.0)	153.2 (97.9–229.2)	146.2 (83.0–223.0)	142.3 (105.0–188.0)	.47
Post-processing of the system (in min) *N* = 159	15.4 (4.3–51.0)	16.0 (5.1–39)	19.1 (5.0–45.0)	13.2 (5.0–23.0)	.21
Non-surgical, patient independent time in total (in min) *N* = 151	94.3 (48.5–143.8)	92.3 (61.3–142.9)	81.6 (42.0–137.4)	68.4 (45.0–179.0)	**< .05**
Total procedure time (in min) *N* = 160	235.2 (149.9–321.3)	232.8 (166.1–321.3)	222.6 (157.0–306.0)	220.8 (170.0–280.0)	.27

*Unconnected *t*-test comparing the Hugo RAS and the daVinci Systems, bolt values indicate reaching of significance at $\alpha = 0.05$

^a^Hugo™ Robotic Assisted Surgery (RAS) System by Medtronic (Medtronic, Minneapolis, MN, USA)

^b^Da Vinci Xi (Intuitive Surgical, Sunnyvale, CA, USA)

^c^Open pelvic procedures; a: Individual procedure count 10–15

A repetition of the above analyses with the full, pre-exclusion dataset, confirms significantly longer NonSPIT (95.92 vs. 81.60 min, *p* = 0.02) and System Preparation (45.86 vs. 32.30 min, *p* < 0.01) in HugoRAS compared to Xi. Slower Approaching and Docking of Hugo RAS is not significant in the full dataset including extreme values (7.91 vs. 4.18 min, *p* = 0.14).

### Learning curve

With an interest in the surgeon-independent NonSPIT, we focused on the learning curve of scrub nurses, operating systems prior to the first incision, during approaching and docking and after undocking. They are mainly responsible of the processes that were identified as more time consuming in HugoRAS than in Xi, namely System Preparation (45.1 vs. 33.5 min; *p* < 0.01) and Approaching and Docking (7.0 vs. 4.7 min; *p* = 0.02).

**Fig. 3:**
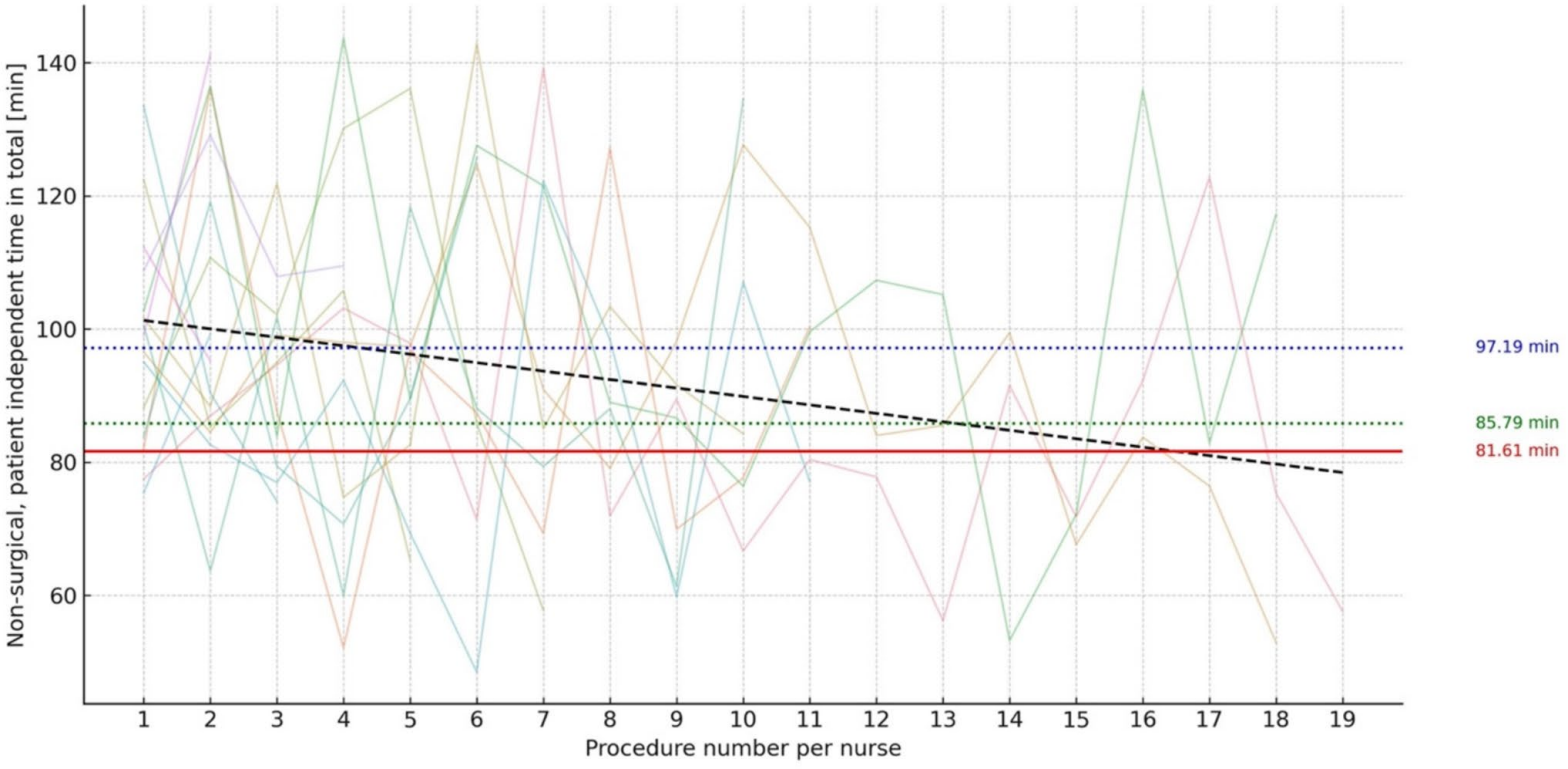
Showing Non-Surgical, Patient Independent Time in min (NonSPIT) per individual procedure count per nurse. Black dotted line: Linear regression of averages of NonSPIT per individual procedure count. Red line: Overall Average NonSPIT in da Vinci Xi^1^ procedures. Blue dotted line: Average NonSPIT of Procedure counts 1–5 in HugoRAS^2^. Green dotted line: Average NonSPIT of Procedure counts 10–15 in HugoRAS^2^; ^1^Da Vinci Xi (Intuitive Surgical, Sunnyvale, CA, USA), ^2^Hugo™ Robotic Assisted Surgery (RAS) System by Medtronic (Medtronic, Minneapolis, MN, USA)

The NonSPIT of unexperienced scrub nurses operating HugoRAS is significantly longer than NonSPIT in Xi (Procedure count 1–5: 97.2 vs. 81.6 min; *p* < 0.01). This disadvantage is eliminated when HugoRAS is operated by experienced scrub nurses (Procedure Count 10–15: 85.8 vs. 81.6 min; *p* = 0.15) (Fig. 3, Table 2).

**Table 2:** Time effects over the course of learning

Time variable	HugoRAS^a^ (*N* = 71)				daVinci Xi^b^ (*N* = 22)
	Unexperienced^c^ (*N* = 43)	p*	Experienced^d^ (*N* = 28)	*p*^+^	
Non-surgical, patient independent time in total (NonSPIT) (in min) *N* = 84	97.2 (52.1–143.75)	**< .01**	85.8 (53.2–134.6)	.15	81.6 (42.0–137.4)
System preparation (in min) *N* = 88	48.0 (26.1–67.2)	**< .01**	44.5 (16.5–65.0)	**< .01**	33.5 (14–54)
Surgical preparation (in min) *N* = 93	20.4 (11.2–36.4)	.69	22.0 (12.5–37.2)	.24	19.8 (11–37)
Approaching and docking (in min) *N* = 87	7.5 (2.0–16.8)	**.01**	6.9 (1.9–17.0)	.06	4.7 (1.1–14)
Undocking and reversing (in min) *N* = 80	4.0 (1.1–12.3)	.98	5.6 (1.4–14.3)	.10	4.0 (1.0–11.0)
Post-processing of the system (in min) *N* = 76	16.6 (5.3–48.0)	.07	16.0 (5.1–39)	.46	19.1 (5.0–45.0)

*Unconnected *t*-test comparing Hugo RAS procedures with unexperienced scrub nurses and the daVinci procedures, ^+^Unconnected t-test comparing Hugo RAS procedures with experienced scrub nurses and daVinci procedures. Bolt values for *p*-values indicate reaching of significance at $\alpha = 0.05$

^a^Da Vinci Xi (Intuitive Surgical, Sunnyvale, CA, USA)

^b^Hugo™ Robotic Assisted Surgery (RAS) System by Medtronic (Medtronic, Minneapolis, MN, USA)

^c^Scrub nurses with individual procedure count 1–5

^d^Scrub nurses with individual procedure count 10–15

An analysis of the NonSPIT components shows a total Delta of 18.8 min between NonSPIT of Xi and unexperienced HugoRAS, of which 77.1% (14.5 min) are caused by differences in System Preparation and 14.9% (2.8 min) by differences in Approaching and Docking.

A repetition of the above analysis with the full, pre-exclusion dataset, confirms a significantly slower NonSPIT in unexperienced HugoRAS staff compared to Xi (103.13 vs. 81.61 min, *p* = 0.01) and the resolution of this difference when comparing experienced HugoRAS staff to Xi (95.87 vs. 81.60 min, *p* = 0.08).

### Estimation of time and costs

Considering the above data, several simulations of cost of lost time are possible.

With an unexperienced team performing surgeries on Hugo, about 18.8 min of patient independent time (NonSPIT) is lost per procedure compared to an Xi (97.2 vs. 81.6 min; *p* = 0.01). This results in a loss of $865.55 in OR time. If compared to OPP (NonSPIT-equivalent: 68.4 min), even 28.8 min, i.e. $1325.95 are lost. Comparing OPP to an established Xi, still 13.2 min or $607.73 are lost.

Consulting the regression line in Fig. 2 with an intercept of 102.56 and a slope of − 1.27, a timely parity between HugoRAS and Xis’ NonSPIT would mathematically occur after 16.5 procedures per nurse, even though statistically, significant time differences is already undetectable after 10–15 procedures per nurse.

The cost occurring during these 16.5 first procedures (Area between black dotted and solid red line) would sum up to 152.43 min or $7017.87. These costs of learning assume that all procedures are carried out by the same scrub nurse, suggesting a multiplication by the size of the team of nurses for a suitable estimate.

The continued disadvantage of prolonged preparation time in experienced HugoRAS staff compared to the Xi (44.5 vs. 33.5, *p* < 0.01) amounts to 9 min or $414.36.

## Discussion

### System comparison

This large sample study on 144 HugoRAS and 23 Xi procedures has confirmed previous, flawed findings of Docking [10] and System Preparation [11] times being the key factors in the timely disadvantage of HugoRAS for non-console time per procedure. System preparation was an average of ca 12 min (45.1 vs. 33.5 min; *p* < 0.01) and Approaching and Docking an average of 2 min (7.0 vs. 4.7 min; *p* = 0.02) longer for HugoRAS. The comparison to the historic OPP reference for preparation time (27.4 min) underlines the higher logistic effort of robotic surgery.

For the first time, this study found no difference in the total procedure time (235.2 vs. 222.6 min; *p* = 0.27) of over 140 HugoRAS procedures and Xi. We were able to show that economically relevant NonSPIT is significantly different between both systems when unexperienced nurses operate the systems (97.2 vs. 81.6; *p* = 0.01) but successfully shrinks below detectability after no more than 10–15 procedures per nurse (85.8 vs. 91.6; *p* = 0.35).

### Learning curve

One comparable previous study analyzing the introduction of HugoRAS in a center previously operating a daVinci system, failed to see a reduction of preoperative times over 30 surgeries, with a remaining disadvantage of ca 25 min [11]. A large ‘residual’ difference, considering our data shows a drawback in NonSPIT of less than 20 min for HugoRAS even during a nurse’s first procedures (97.2 vs. 81.6; *p* = 0.01). That is especially distinctive as the previous study started recording the time with the patient’s arrival inside the OR [11], i.e. later than implemented in our study. Our findings consolidate HugoRAS as a time-economically equivalent alternative to the Xi system, when building on preexisting daVinci experience and allowing for a personal learning curve of 10–15 procedures.

The average Approaching and Docking time for HugoRAS in this study (inexperienced HugoRAS: 7.5 min vs. experienced Hugo: 6.9 min vs. Xi: 4.5 min) shows a long-stretched learning curve for optimization of the multi-cart process. Previous data from this group published in 2024 emphasized the importance of scrub nurse experience in docking. It showed an average docking time of 13.5 min over 56 procedures [10], while other groups needed an average of 11 min over 50 cases [19] and 10 min over 132 cases [20]. The furthermore reduced docking time in this study underlines the importance of continued experience to speed up docking. In this group’s workflow, docking is carried out by both console surgeon and bedside surgeon simultaneously, while the unsterile scrub nurse approaches the carts.

It is common knowledge that surgical time, including console time, still amounts for the largest portion of procedure time. However, this study also clarified that NonSPIT accounts for an immensely relevant 37–40% (daVinci: NonSPIT: 81.6 min, Total Procedure Time: 222.6 min; HugoRAS: NonSPIT: 94.3 min, Total Procedure Time: 235.2 min) of Total Procedure Time. In face of demographically rising RARP patient numbers [1], declining availability of nursing staff [12] and its rather indirect effect on treatment, reducing NonSPIT should be a key joint objective of manufacturers and OR managements. Shortening NonSPIT would leave more room for surgical time or even allow for additional procedures per day. This study showed that teams are able to reduce the differences in NonSPIT of HugoRAS and Xi past the level of significance (85.8 vs. 81.6; *p* = 0.15).

Interestingly, System Preparation, the largest NonSPIT component (HugoRAS: 48%, Xi: 41%) is still relevantly higher in Medtronic’s system than in Intuitive’s after 15 procedures (44.5 min vs. 33.5 min; *p* < 0.01).

### Cost implications

The simulated cost of 152.43 min or $7017.87 needed per nurse starting to operate HugoRAS to reach the Xi-known level of efficiency in NonSPIT may be seen as an initial investment to establish a different robotic system. The remaining 9 min or $414.36 disadvantage in preparation time, however, jeopardize the cost savings of approximately 250€ (USD equivalent at publication: $270) per procedure that were previously reported [13]. Naturally, the financial estimations are only an approximation to a system of very individual prices and costs for systems, services, times and personnel. Their generalizability from a German tertiary care center to the US market, where HugoRAS is about to be introduced, is limited. The temporal differences can be used as an approximation but must be set into the context of local conditions.

### Limitations

Our study faces several limitations. Firstly, the number of procedures differed widely for both systems. However, the main observations were made in the dynamic Hugo cohort, where a learning curve was to be expected and studied within this cohort. As the daVinci system had been in place for > 15 years, and OPP data stems from a time of high OPP caseload, processes were very standardized and the latter data serves as a constant, reliable average reference to new HugoRAS data. Thus, we prioritized data and resource efficiency in accordance with data privacy considerations of the ethics approval. A propensity score matching had been discussed but was ultimately rejected due to limited and methodologically unsuitable confounders leading to problems of overmatching. The assumption of a linear development and reaching of the Xi’s level is yet to be confirmed and only to be used as an estimation.

Secondly, randomization of staff was operationally out of reach, as each nurse new to HugoRAS had to work with one having performed several procedures. All nursing staff included was experienced in RAS with years of Xi procedures. This fact inhibited deeper insights on adaption of HugoRAS as a first robotic system. As the focus of this study lies on non-surgical and times patient-independent organizational times, the study’s data collection design did not include relevant patient- or surgeon-specific confounders. Even though procedure count was recorded for the surgeon involved and each team configuration, analyzing or controlling for these variables is of limited validity due to the surgeons’ incoherent allocation to one or both systems and the overwhelmingly high number of possible team compositions.

Due to the high number of Nurses involved (21), not all nurses reached a procedure count of 10–15 (Measurements for procedure numbers 10–15: *N* = 24). This lowers the power of the analysis but matches the reality of most urologic departments with rotating scrub nurses.

Overall, measuring time periods in a working environment by human input is subject to inaccuracies as transparently visible in the unaccounted time in Fig. 2. However, this mainly occurred for OPP due to retrospective acquisition and was thereby mainly excluded from statistical analysis with minimal relevance to the metric of NonSPIT in RPP (Unaccounted Time HugoRAS: 1.4% of NonSPIT, Xi: 0.6% of NonSPIT).

In summary, the above numbers can form an approximation for an economical and administrative decision before acquiring new robotic system. Even though no recent data is publicly available, cost of an U.S.-American OR minute varied between $25–$133/min in pre-pandemic years [14–16], recently averaging at $46 [17]. Similarly, the cost of different robotic systems for a department widely varies depending on pricing models, financing or shared usage. However, this study shows that an average of 152 min additional NonSPIT (NonSPIT HugoRAS vs. Xi: 97.2 vs. 81.6; *p* = 0.01) is needed over the course of about 16 procedures per nurse to cancel out the overall adaption phase of HugoRAS as a second robotic system. We call for further streamlining of system preparation times as an inertly improving driver of NonSPIT. Although this study gives an approximation of additional organizational cost, the implementation of new robotic systems must obviously focus on clinical outcome data that can merely be flanked by economical observations.

## Conclusion

Non-surgical, patient independent time takes up approximately 40% of total procedure time in RAS of the pelvis and OR time is precious. When HugoRAS is introduced as a secondary robotic system it is initially subject to a time disadvantage, which pre-experienced staff can even out after 10–15 procedures, leaving a minor disadvantage in system preparation.

## Supplementary Information

Below is the link to the electronic supplementary material.

Supplementary file1 (DOCX 17 KB)

## Data Availability

The data that support the findings of this study are not openly available due to reasons of sensitivity and are available from the corresponding author upon reasonable request.
