# Supplementary material for: Impact of non-surgical OR time on efficiency and costs with Hugo™ RAS
Source: J Robot Surg. 2025 Sep 5;19(1):565. doi: 10.1007/s11701-025-02731-5 (PMC12413327; doi:10.1007/s11701-025-02731-5)
Supplement: Supplementary file 1 — Supplementary file1 (DOCX 17 KB) [file 11701_2025_2731_MOESM1_ESM.docx]

# **Supplements**

**Supplementary Table 1**

| Total procedures  N = 187 | **Missing Values** | **Available values** | **Excluded by SD** | **Excluded by min. value** | **Valid**  **values** | **Exclusion**  **Total** |
| --- | --- | --- | --- | --- | --- | --- |
| System Preparation | 0 | 187 | 10 | - | **177** | 10 |
| Surgical Preparation | 0 | 187 | 6 | - | **181** | 6 |
| Incision and Trocar Placement | 24 | 163 | 4 | 10 | **149** | 38 |
| Approaching and Docking | 26 | 161 | 1 | 4 | **156** | 31 |
| Undocking and Reversing | 23 | 164 | 9 | 12 | **143** | 44 |
| Incision to Suture | 13 | 174 | 6 | - | **168** | 19 |
| Post-Processing of the System | 3 | 184 | 8 | 17 | **159** | 28 |
| Non-Surgical, Patient Independent Time in total | 32 | 155 | 4 | - | **151** | 36 |
| Total Procedure Time | 19 | 168 | 8 | - | **160** | 27 |
